# Catamenial haemolacria and ocular pain as rare manifestations of extra-pelvic endometriosis

**DOI:** 10.1093/jscr/rjag078

**Published:** 2026-10-12

**Authors:** Emine Karabük, Beste Çakmakgil, Ayberk Çakır, Mehmet Faruk Köse

**Affiliations:** Department of Obstetrics and Gynecology, Acıbadem Mehmet Ali Aydınlar University, School of Medicine, 34303, Istanbul, Turkey; Acıbadem Mehmet Ali Aydınlar University, School of Medicine, Istanbul; Department of Obstetrics and Gynecology, Acıbadem Atakent University, 34303, Istanbul, Turkey; Department of Obstetrics and Gynecology, Acıbadem Mehmet Ali Aydınlar University, School of Medicine, 34303, Istanbul, Turkey

**Keywords:** ocular endometriosis, catamenial haemolacria, extrapelvic endometriosis, adolescent gynecology, hormonal therapy, dienogest

## Abstract

Ocular involvement in endometriosis is an exceptionally rare extrapelvic manifestation, typically presenting as catamenial haemorrhagia. We report the case of an adolescent girl who presented with recurrent eye bleeding and pain during her menstruation. A comprehensive physical examination and imaging, including orbital magnetic resonance imaging, were unremarkable. Hematologic and ophthalmological evaluations excluded coagulopathies and structural ocular pathologies. Given the cyclical pattern of her symptoms and the absence of other identifiable causes, a presumptive diagnosis of ocular endometriosis was made. The patient was treated with a combination of dienogest and ethinyl estradiol, leading to significant symptom improvement over a two-year follow-up period. Occasional minimal haemolacria persisted, but no further intervention was required. This case underscores the importance of considering endometriosis in the differential diagnosis of catamenial haemolacria, even in the absence of radiological confirmation. A multidisciplinary approach and individualized hormonal therapy can result in substantial symptom control and improved quality of life in affected patients with catamenial haemolacria.

## Introduction

Endometriosis is a chronic inflammatory disease characterized by the presence of endometrial-like tissue outside the uterine cavity, affecting approximately 10% of women of reproductive age [1, 2]. While it commonly involves pelvic structures such as the ovaries, peritoneum, and uterosacral ligaments, extrapelvic locations, including the gastrointestinal and urinary systems, are also recognized, although less frequently [3–5]. Diagnosis of extra-pelvic endometriosis remains challenging due to nonspecific symptoms and low clinical suspicion, often resulting in delayed treatment and reduced quality of life [6].

Although uncommon, ocular involvement may occur and typically presents with catamenial haemolacria—cyclical bloody tearing during menstruation—which can significantly impact the physical and psychosocial well-being [7–9]. Given its rarity, such presentations may be misattributed to other systemic or hematologic disorders due to its rarity.

In this case report, we present an adolescent patient with presumed ocular endometriosis who experienced cyclical haemolacria and pain. This study emphasizes the importance of considering endometriosis in atypical clinical settings and illustrates a multidisciplinary diagnostic and therapeutic approach.

## Case report

We report the case of a female patient in her early teens who presented to our outpatient clinic with cyclical ocular bleeding that coincided with her menstruation (Fig. 1) (Video 1). Her symptoms included pain in the inguinal region, periorbital area, lips, and umbilicus. Menarche had occurred approximately one year prior, but her menstrual cycle had never been regular.

**Figure 1:**
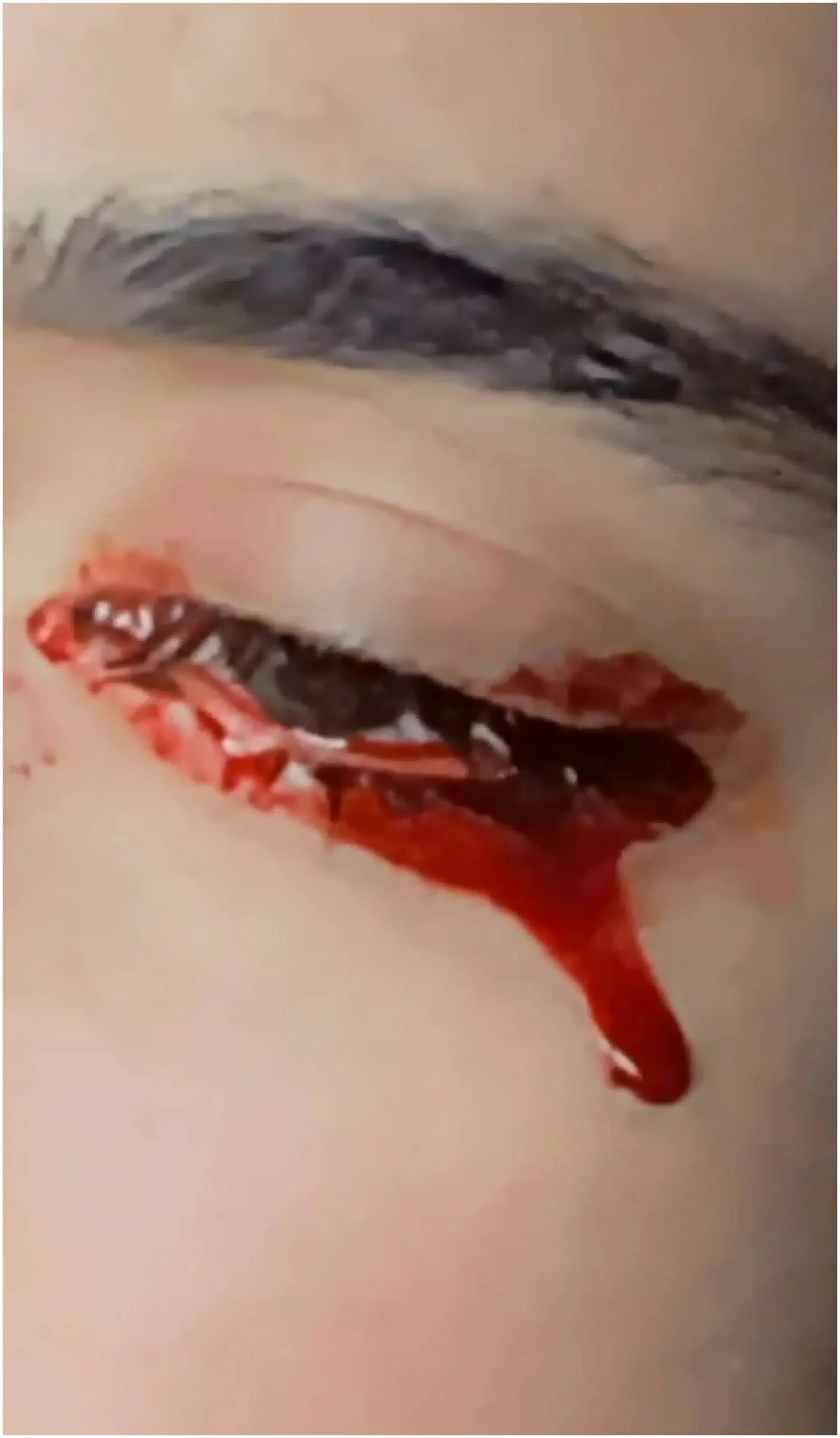
Catamenial haemolacria observed during menstruation in the patient. The image shows blood droplets at the medial canthus of the left eye. No ocular pathology was identified during the ophthalmological examination.

The patient had no known chronic conditions, did not regularly use any medication, and had no prior surgical history. On physical examination, the abdomen was soft and non-tender, with no palpable masses or organomegaly. Transabdominal pelvic ultrasonography revealed a uterus of normal size and echogenicity for her age at presentation. Both ovaries displayed normal volumes and follicular distributions, and no intra-abdominal fluid or adnexal masses were observed. The patient provided photographic and video evidence of haemolacria during menstruation, manifesting as episodic blood-tinged tears. The patient was referred to the ophthalmology and hematology departments for further evaluation.

Comprehensive laboratory testing, including a thrombophilia panel, von Willebrand factor, platelet function assays, iron studies, international normalized ratio, and complete blood count, yielded results within the normal limits. Ophthalmological assessment, including biomicroscopy, funduscopy, and visual acuity testing, revealed no abnormalities. Orbital magnetic resonance imaging was performed to evaluate potential lacrimal system pathology or vascular malformations; however, no structural lesions were identified.

Given the temporal correlation with the menstrual cycle and exclusion of other etiologies, a presumptive diagnosis of ocular endometriosis was made. Treatment was initiated with 2 mg/day of dienogest and a combined oral contraceptive containing 0.03 mg of ethinyl estradiol. At two-year follow-up, the patient demonstrated marked clinical improvement, reporting minimal menstrual pain and a significant reduction in ocular bleeding episodes. While occasional minor haemolacria persisted during select cycles, the symptoms were substantially diminished. Ongoing management consisted of continued dienogest therapy, which was later transitioned to a combination of dienogest and estradiol valerate, with plans to maintain this regimen until conception was achieved. Further follow-up was not recommended by the ophthalmology or hematology team.

## Discussion

We report a case of an adolescent with ocular manifestations of endometriosis, presenting with catamenial haemolacria and pain in the absence of detectable ocular pathology on examination or imaging findings. Ocular endometriosis is an exceptionally rare manifestation and one of the rarest causes of recurrent hemorrhage in the eye. Owing to anatomical constraints, histological confirmation via biopsy is often unfeasible, making clinical diagnosis challenging [10]. Despite its rarity, endometriosis should not be overlooked, as delayed diagnosis can lead to long-term complications, including chronic pelvic pain, infertility, and a slightly increased risk of ovarian, breast, and thyroid malignancies [11].

A thorough differential diagnosis is essential for evaluating hematolacria. Potential causes include infections, lacrimal sac mucoceles, vascular malformations, systemic hematologic disorders, and malignancies [12]. In this case, extensive hematological and ophthalmological evaluations were unremarkable. The cyclic nature of the symptoms, correlating with menstruation, and the exclusion of other etiologies supported the presumptive diagnosis of ocular endometriosis, even in the absence of radiological or histopathological evidence of disease.

The pathophysiology of extrapelvic endometriosis remains incompletely understood, with proposed mechanisms including retrograde menstruation, hematogenous or lymphatic dissemination, coelomic metaplasia, and hormone-regulated inflammatory responses [13]. Given the wide variability in clinical presentation and the multifactorial nature of its etiology, standardized treatment approaches are currently lacking. A multidisciplinary strategy is often recommended, involving collaboration with hematology, general surgery, thoracic surgery, and other specialties when necessary [5].

Hormonal suppression is the cornerstone of the treatment. Combined oral contraceptives and progestins are preferred as first-line therapies, whereas gonadotropin-releasing hormone analogs are reserved for second-line use [9, 10, 14]. Surgical management is not routinely advised as the initial therapy, and treatment plans should be individualized based on symptom severity, treatment response, fertility desires, and lesion location [11, 13, 15].

It is also important to emphasize the under-recognition of endometriosis in the adolescent population. The condition frequently presents with normalized or dismissed symptoms, such as dysmenorrhea and chronic pelvic pain, leading to diagnostic delays and a detrimental impact on quality of life [14].

A limitation of this case report is the lack of histopathological confirmation. However, this is a common challenge in extrapelvic endometriosis cases. In our patient, the temporal association with menstruation, characteristic symptoms, and favorable response to hormonal therapy collectively supported this diagnosis.

Ocular endometriosis, although rare, should be considered in women of reproductive age who present with cyclical haemolacria. Early diagnosis and individualized hormonal therapy can offer significant symptom relief and prevent long-term disease sequelae. This case underscores the importance of multidisciplinary collaboration in managing atypical endometriosis presentations.

## Supplementary Material

Video_1_rjag078

Video_1_rjag078

## References

[1] Pašalić E, Tambuwala MM, Hromić-Jahjefendić A. Endometriosis: classification, pathophysiology, and treatment options. Pathol-Res Practice 2023;251:154847. 10.1016/j.prp.2023.154847 37844487

[2] Zondervan KT, Becker CM, Kago K et al. Endometriosis. Nat Rev Dis Primers 2018;4:9. 10.1038/s41572-018-0008-5 30026507

[3] Lee HJ, Park YM, Jee BC et al. Various anatomic locations of surgically proven endometriosis: a single-center experience. Obstet Gynecol Sci 2015;58:53. 10.5468/ogs.2015.58.1.53 PMC4303753 25629019

[4] Chauhan S, More A, Chauhan V et al. Endometriosis: a review of clinical diagnosis, treatment, and pathogenesis. Cureus 2022;14:e28864. 10.7759/cureus.28864 PMC9537113 36225394

[5] Hirata T, Koga K, Osuga Y. Extra-pelvic endometriosis: a review. Reprod Med Biol 2020;19:323–33. 10.1002/rmb2.12340 PMC7542014 33071634

[6] Shim JY, Laufer MR. Adolescent endometriosis: an update. J Pediatr Adolesc Gynecol 2020;33:112–9. 10.1016/j.jpag.2019.11.011 31812704

[7] Griffiths MJ, Horne AW, Gibson DA et al. Endometriosis: recent advances that could accelerate diagnosis and improve care. Trends Mol Med 2024;30:875–89. 10.1016/j.molmed.2024.06.008 38991858

[8] International working group of AAGL E, ESHRE, WES, Tomassetti C, Johnson NP et al. An international terminology for endometriosis, 2021. Hum Reprod Open 2021;2021:hoab029. 10.1093/hropen/hoab029 PMC8530702 34693033

[9] Rahman S, Youssef Y, Maroun G et al. Eyes, menstruation and endometriosis. Facts, Views & Vision in ObGyn 2023;15:107. 10.52054/FVVO.15.2.074 PMC10410658 37436046

[10] Celebi ARC, Aygun EG. A rare cause of recurrent subconjunctival hemorrhage: ocular vicarious menstruation. GMS Ophthalmology Cases 2023;13:Doc05. 10.3205/oc000213 PMC9979076 36875630

[11] Becker CM, Bokor A, Heikinheimo O et al. ESHRE guideline: endometriosis. Hum Reprod Open 2022;2022:hoac009. 10.1093/hropen/hoac009 PMC8951218 35350465

[12] Kaushik M, Juniat V, Ezra DG et al. Blood-stained tears—a red flag for malignancy? Eye 2023;37:1711–6. 10.1038/s41433-022-02224-x PMC10220060 36088421

[13] Cano-Herrera G, Salmun Nehmad S, de Chávez R et al. Endometriosis: a comprehensive analysis of the pathophysiology, treatment, and nutritional aspects, and its repercussions on the quality of life of patients. Biomedicines 2024;12:1476. 10.3390/biomedicines12071476 PMC11274817 39062050

[14] Shim JY, Laufer MR, King CR et al. Evaluation and management of endometriosis in the adolescent. Obstet Gynecol 2024;143:44–51. 10.1097/AOG.0000000000005448 37944153

[15] Alonso A, Gunther K, Maheux-Lacroix S et al. Medical management of endometriosis. Curr Opin Obstet Gynecol 2024;36:353–61. 10.1097/GCO.0000000000000983 PMC11356688 39159261

