# Supplementary material for: Catamenial haemolacria and ocular pain as rare manifestations of extra-pelvic endometriosis
Source: J Surg Case Rep. 2026 Oct 12;2026(10):rjag078. doi: 10.1093/jscr/rjag078 (PMC13657181; doi:10.1093/jscr/rjag078)
Supplement: Supplementary file 1 [file video_1_rjag078.docx]

**Video 1.** Video footage recorded by the patient during menstruation, demonstrating haemolacria in the left eye. The bleeding was cyclical and coincided with menstrual episodes
